# Laparoscopic repeat hepatectomy versus conventional open repeat hepatectomy for recurrent hepatocellular carcinoma: A systematic review and meta-analysis

**DOI:** 10.3389/fonc.2022.960204

**Published:** 2022-09-15

**Authors:** Fulong Hao, Hancong Li, Nan Li, Jiaxin Li, Hong Wu

**Affiliations:** ^1^ Department of Liver Surgery and Liver Transplantation Centre, State Key Laboratory of Biotherapy and Cancer Center, West China Hospital, Sichuan University, Chengdu, China; ^2^ Department of Hepatobiliary Surgery, Suining First People’s Hospital, Suining, China; ^3^ West China School of Medicine, West China Hospital, Sichuan University, Chengdu, China; ^4^ Engineering Research Centre of Medical Information Technology, Ministry of Education, West China Hospital, Sichuan University, Chengdu, China; ^5^ Information Technology Centre, West China Hospital of Sichuan University, Chengdu, China

**Keywords:** recurrence, hepatocellular carcinoma, laparoscopic repeat hepatectomy, open repeat hepatectomy, meta-analysis

## Abstract

**Background:**

Repeat hepatectomy has been proven to be an effective treatment in patients with recurrent hepatocellular carcinoma (RHCC). However, for RHCC, it is still controversial whether laparoscopic hepatectomy is superior to conventional ones. The present meta-analysis was carried out to investigate the safety and overall effect of laparoscopic repeat hepatectomy (LRH) to open repeat hepatectomy (ORH) for patients with RHCC.

**Methods:**

A meta-analysis was registered at PROSPERO, and the registration number is CRD42021257569. PubMed, Web of Science, and EMBASE were searched based on a defined search strategy to identify eligible studies before 25 April 2022. Data on operative times, bleeding volume, overall complications, 90-day mortality, blood transfusion, length of stay, overall survival rate, and long-term recurrence-free survival rate were subjected to meta-analysis.

**Results:**

Overall, we identified nine studies of LRH versus ORH enrolling a total of 945 patients (460 and 485 underwent LRH and ORH, respectively). The present meta-analysis revealed non-significant differences in operative time, blood transfusion, overall complications, 90-day mortality, 3-year overall survival rate, 5-year overall survival rate, and long-term recurrence-free survival rate between the two groups. Alternatively, comparing LRH with ORH, LRH has less bleeding volume (p < 0.001) and a shorter length of stay (p = 0.005).

**Conclusion:**

LRH is a feasible and effective treatment strategy for RHCC.

**Systematic review registration:**

https://www.crd.york.ac.uk/PROSPERO/#searchadvanced, identifier CRD42021257569.

## 1 Introduction

Liver cancer is the third leading cause of cancer-related death worldwide and ranks sixth in terms of morbidity (1). Hepatocellular carcinoma (HCC) accounts for 75% to 95% of all primary liver cancers (2). Due to its rising incidence and unfavorable prognosis, HCC was considered a major global health problem (3). Hepatectomy has long been the frequent curative treatment for HCC and is especially appropriate for patients at an early stage (4–6). Unfortunately, tumor recurrence occurred in as many as 60%–80% of cases at 5 years, which made the long-term outcomes of HCC to remain unsatisfactory (6–9). No accepted neoadjuvant or adjuvant therapies have been confirmed to reduce the risk of recurrence (6, 7, 10). Hence, an effective therapeutic regimen for recurrence is essential to prolonging survival for HCC patients (11, 12).

Currently, varieties of remedies including repeated hepatectomy, liver transplantation, embolization, ablation, and molecular targeted therapy have been widely used in the clinical treatment of recurrent hepatocellular carcinoma (RHCC) (11, 13, 14). However, guidelines for the management of RHCC remained controversial (11, 12). Multiple studies have endorsed repeat hepatectomy as an effective treatment with favorable long-term surgical outcomes for RHCC in the past few decades (15–17).

Previous operation history had been among the contraindications for laparoscopic surgery (18). Nevertheless, with the improvement of laparoscopic instruments and accumulation of surgical techniques, laparoscopic hepatectomy (LH) has emerged as a viable alternative treatment to open hepatectomy (OH) and has been applied in specific RHCC patients safely (19, 20). Previous literature has confirmed the safety and efficiency of LH, emphasizing that LH was superior to OH due to less bleeding volume, shorter operation time, and faster recovery (21, 22).

However, postoperative adhesions as well as changes in anatomical land marks and liver deformation may cause technical challenges for laparoscopic repeat hepatectomy (LRH). The indication criteria for LRH have yet to be clearly defined (23). Hence, whether LRH or ORH is the preferred treatment for RHCC remains elusive.

To address this issue, we conducted a meta-analysis to compare the clinical efficacy and safety of LRH and ORH for patients with RHCC.

## 2 Methods

This study was carried out following the PRISMA 2020 guideline (24). The protocol of the present review was registered and allocated the identification number CRD42021257569 in the PROSPERO database.

### 2.1 Search strategy and study selection

Published documents before 25 April 2022 were retrieved using the electronic databases PubMed, EMBASE, Web of Science, and Cochrane Central Register, by two independent researchers (FL Hao, HC Li). The following subject terms were employed in the literature search: recurrent liver cancer, recurrent hepatocellular carcinoma, laparoscopic hepatectomy, open hepatectomy, liver resection, and minimally invasive surgery. **Supplementary Table S1** shows our search strategy. For gaining additional trials, a manual search of eligible studies in references was complemented.

### 2.2 Inclusion and exclusion criteria

Two researchers (FL Hao, HC Li) identified and reviewed full-text articles that were regarded as relevant by screening the titles and abstracts. Disagreements were resolved by a team discussion.

Inclusion criteria were as follows (1): participants—patients with RHCC after initial curative liver resection (2); types of interventions—LRH and ORH (3); data available on interesting surgical outcomes.

Exclusion criteria were as follows (1): The publication type was observational clinical studies, case–control studies, abstracts, editorials, case reports, letters, and expert opinion (2); studies without available data, non-English or experimental studies.

### 2.3 Data extraction

Two researchers (FL Hao, HC Li) independently extracted relevant data with a standardized form. The data from studies based on a PSM analysis were extracted from the post-PSM analysis. Any ambiguity was discussed with the third researcher (N Li).

Based on the predetermined criteria, the following data were extracted: name of the first author, publication year, study design, country, number of patients, mean age, gender, tumor size, tumor number, operative times, bleeding volume, blood transfusion, number of patients converted from laparoscopy to laparotomy, overall complication, hospitalization, 90-day mortality, 1-, 3-, and 5-year survival (OS) rate, and 1-, 3-, and 5-year recurrence-free survival (RFS) rate.

### 2.4 Quality assessment

The Newcastle–Ottawa Scale (NOS) developed for evaluating the quality of eligible studies was utilized by two independent reviewers (FL Hao, HC Li) (24). NOS score ≥6 was defined as high-quality. Any disagreements were discussed and resolved through consensus.

### 2.5 Statistical analysis

Statistical analysis was performed using the Review Manager software (RevMan V.5.3.4). Continuous data were expressed as 95% confidence interval (CI) and mean difference (MD), while dichotomous data used odds ratio (OR). For overall survival data, we used Engauge Digitizer (RevMan V.4.1) to extract OS and RFS data from survival curves (25). Using the method originally described by Hozo et al., medians with ranges were converted into means with standard deviations (26). Publication bias was assessed *via* Begg’s funnel plot and Egger’s linear regression test. Heterogeneity was examined by the I^2^ statistic. Statistical heterogeneity is significant when I^2^ ≥50%, and the random-effect model (REM) is utilized; if not (I^2^ <50%), the fixed-effect model (FEM) is applied.

## 3 Results

### 3.1 Literature search results

**Figure 1:**
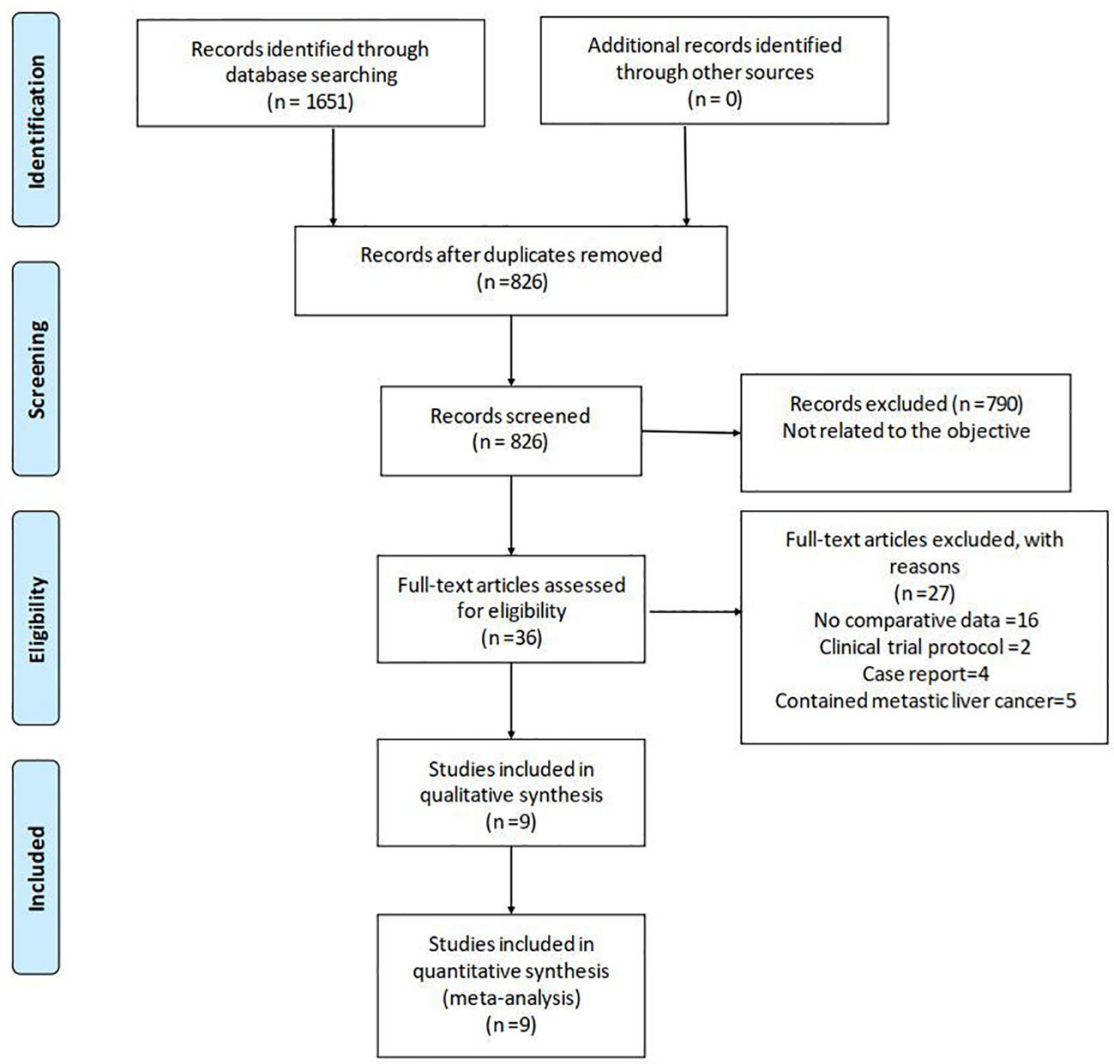
Flowchart of study identification and selection.

The literature search yielded 1,651 relevant English publications which were considered potential studies. Eight hundred twenty-five of them were duplicates. Seven hundred ninety articles were excluded for irrelevance to the objective after screening the abstract and partial full text. Thirty-six full-text articles met the eligibility for assessment. Through reading the full text, 27 studies were excluded due to inappropriate study design or content. Finally, according to the inclusion criteria, nine studies (23, 27–34) of a total of 945 patients (460 and 485 underwent LRH and ORH, respectively) were found to be eligible for the present meta-analysis. **Figure 1** shows the procedure of study selection in a flow diagram. Detailed NOS scores are presented in **Supplementary Table S2**.

### 3.2 Characteristics of the included studies

In this review, we included nine studies involving 945 patients. The overall characteristics of the included articles are shown in **Table 1**. The sample sizes varied from 33 to 476, and most study designs were PSM studies.

**Table 1:** The main characteristics of the included studies in this meta-analysis.

Author year	Country	Study design	Period	Patients		Age (year)		Gender (M/F)		Tumor size (cm)		Pathology	No. of tumors		No. of conversion	Child-Pugh Score (A/B)		Previous operation (open/LH)		Etiology		BCLC stage		Tumor grading		Tumor location		Co-morbid illness		Resection margin		
				LH (n)	OH (n)	LH	OH	LH	OH	LH	OH		LH	OH		LH	OH	LH	OH	LH	OH	LH	OH	LH	OH	LH	OH	LH	OH	LH	OH	
Kanazawa-2013	Japan	RM	2006-2011	20	20	70 (46–83)	65 (43–74)	19/1	15/5	1.7 (0.7–3.5)	2.2 (1.3–4.1)	HCC	Solitary: 16Multiple:4	Solitary: 18Multiple:2	2	19/1	17/3	15/5	NA	HBV:4HCV:11NBNC:5	HBV:6HCV:10NBNC:4	NA	NA	NA	NA	Segments II, III, IV, V, VI: 13Segments VII, VIII:5Segments I:1Biober:1	Segments II, III, IV, V, VI: 13Segments VII, VIII: 4Segments I:2Biober:1	NA	NA	NA	NA	
Chan-2014	China	Case-match	2004-2013	11	22	61(43-80)	62(43-76)	8/3	16/6	2 (1.0–4.5)	2 (1.0–5.0)	HCC	Solitary: 10Multiple:1	Solitary: 20Multiple:2	0	11/0	22/0	6/5	NA	HBV:7HCV:1	HBV:18HCV:2	NA	NA	NA	NA	Left lobe: 7Right lobe: 4	Left lobe: 14Right lobe: 8	Cardiovascular: 3Respiratory: 1	Cardiovascular: 6Respiratory:1Diabetes mellitus: 2Gastrointestinal: 2	Not involved: 11Involved: 0	Not involved: 20Involved: 2	
Zhang-2016	China	P	2014-2014	31	33	54 (37–66)	59.5 (34–65)	26/5	27/6	2.5 ± 1.0	3.8 ± 1.1	HCC	NA	NA	0	31/0	33/0	31/0	33/0	NA	NA	NA	NA	NA	NA	Left lobe: 15Right lobe: 16	Left lobe: 14Right lobe: 19	NA	NA	2.1 ± 1.2	2.2 ± 0.6	
Liu-2017	China	PSM	2008-2015	30	30	56.5 (27–79)	48.5 (28–79)	23/7	28/2	2.1 (1.0–5.0)	2.45 (1.0–4.3)	HCC	Solitary: 25Multiple:5	Solitary: 28Multiple:2	4	30/0	27/3	21/9	NA	HBV:29	HBV:29	NA	NA	NA	NA	Segments II, III, IVa, V, VI:18Segments IVb, VII, VIII:4Segments I:1Biober:7	Segments II, III, IVa, V, VI:15Segments IVb, VII, VIII:8Segments I:3Biober:4	Bile leak: 1Intra-abdominal hemorrhage:1Abdominal infection: 0Ascites:0Liver failure 0	Bile leak: 3Intra-abdominal hemorrhage:1Abdominal infection: 4Ascites:1Liver failure 1	Involved:30	Involved:30	
Goh-2018	Singapore	PSM	2015-2017	20	20	68.5 (67.0–71.75)	69 (63.0–72.25)	18/2	18/2	2 (1.15–2.78)	2.6 (1.5–3.0)	HCC	Solitary: 19Multiple:1	Solitary: 18Multiple:2	3	NA	NA	7/13	NA	HBV: 10	HBV: 10	NA	NA	NA	NA	NA	NA	NA	NA	Resection margin <1 mm: 1	Resection margin <1 mm: 3	
Onoe-2019	Japan	R	2007-2018	30	42	70.9 (50–85)	72.0 (59–88)	23/7	30/12	1.25 (0.08–3.5)	1.75 (0.5–6.0)	HCC	1 (1-3)	1 (1-4)	2	30/0	34/8	21/9	36/6	HBV: 10HCV: 14NBNC: 6	HBV: 13HCV: 17NBNC: 12	NA	NA	NA	NA	Segments VII, VIII: 12Others: 18	Segments VII, VIII: 15Others: 27	NA	NA	NA	NA	
Morise-2020	Japan	PSM	2007-2017	238	238	67.1 ± 11.8	66.4 ± 10.2	181/57	184/54	2.75 ± 2.88	2.77 ± 2.64	HCC	1.28	1.32	NA	NA	NA	181/57	187/51	NA	NA	NA	NA	NA	NA	Anterolateral: 171Posterosuperior:67	Anterolateral: 181Posterosuperior: 57	Ascites: 238Encephalopathy: 238Varices: 238	Ascites: 238Encephalopathy: 238Varices: 238	NA	NA	
Gon-2020	Japan	PSM	2008-2019	23	23	72 (67–79)	72 (67–79)	18/5	18/5	1.9 (1.2–2.5)	2.0 (1.3–2.6)	HCC	Solitary: 22Multiple:1	Solitary: 23Multiple:0	2	23/0	23/0	21/2	23/0	HBV/HCV: 15NBNC: 8	HBV/HCV: 21NBNC: 2	NA	NA	NA	NA	Segments I, VII, VIII: 8Segments II-VI: 15	Segments I, VII, VIII: 9Segments II-VI: 14	NA	NA	NA	NA	
Chen-2021	China	PSM	2017-2018	57	57	56 (36-78)	59 (34-77)	49/8	50/17	1.5 (0.6-4.5)	1.7 (0.8-4.5)	HCC	1 (1-4)	1 (1-2)	6	57/0	57/0	50/7	52/5	HBV: 52	HBV: 53	NA	NA	NA	NA	Anterolateral: 43Posterosuperior: 14	Anterolateral: 47Posterosuperior: 10	NA	NA	NA	NA	

LH, laparoscopic hepatectomy; OH, open hepatectomy; M/F, male/female; PSM, propensity score matching. NA, not applicable.

### 3.3 Operative outcomes

#### 3.3.1 Operative time

**Figure 2:**
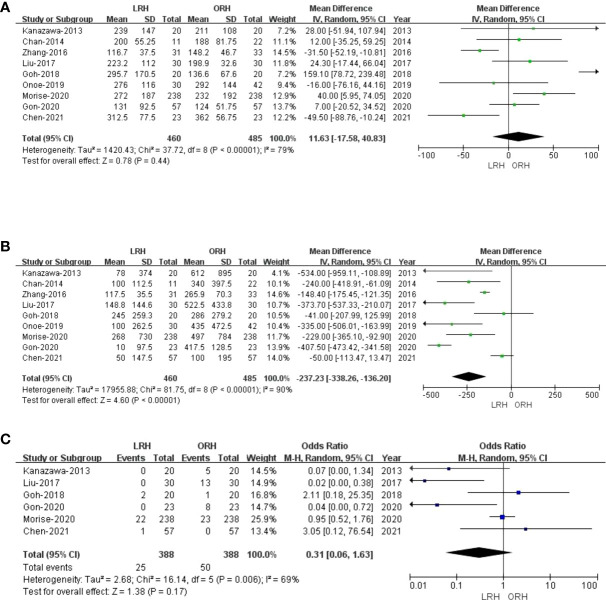
Forest plot of comparison of LRH versus ORH for operative outcomes of survivors.**(A)**, Forest plot for operative time; **(B)**, Forest plot for bleeding volume; **(C)**, Forest plot for blood transfusion.

All of the nine included studies made a comparative evaluation of operative times. Our analysis showed that the operative time in LRH patients was not inferior to those of ORH (MD: 11.63 min; 95% CI: -17.58 to 40.83; p = 0.44). Heterogeneity was high (I^2^ = 79%) and analyzed in the REM (**Figure 2A**).

#### 3.3.2 Bleeding volume

Nine studies that comprised 945 patients (460 and 485 underwent LRH and ORH, respectively) had reported the bleeding volume. Compared with the ORH group, the bleeding volume was lesser in the LRH group (MD: -237.23 ml; 95% CI: -338.26 to -136.20; p<0.00001). Heterogeneity was high (I^2^ = 90%) and analyzed in the REM (**Figure 2B**). A summary of meta-analysis results can be found in **Table 2**.

**Table 2:** Summary results of the meta-analyses.

Outcomes of interest	Studies, n	LRH	ORH	MD/OR (95% CI)	P value	Heterogeneity				Evidence quality
						X^2^	df	I^2^, %	P value	
**Operative outcomes**										
Operative time	9	460	485	11.63 (-17.58,40.83)	0.44	37.72	8	79	<0.00001	Low
Bleeding volume	9	460	485	-237.23 (-338.26, -136.20)	<0.00001	81.75	8	90	<0.00001	Very low
Blood transfusion	6	388	388	0.31 (0.06, 1.63)	0.17	16.14	5	69	0.006	Low
**Postoperative outcomes**										
Overall complication rates	8	429	452	0.44 (0.17, 1.14)	0.09	19.29	7	64	0.007	Low
Length of stay	9	460	485	-2.52 (-4.27, -0.76)	0.005	55.68	8	86	<0.00001	Low
90-Day mortality	4	308	308	1.00 (0.25, 4.06)	1.00	1.96	2	0	0.37	Moderate
**Oncological outcomes**										
1-year overall survival rate	3	279	290	0.60 (0.41,0.89)	0.01	2.50	2	20	0.29	Moderate
3-year overall survival rate	2	268	268	1.06 (0.31,3.62)	0.93	3.30	1	70	0.07	Low
5-year overall survival rate	2	268	268	0.76 (0.44,1.32)	0.33	1.44	1	31	0.23	Moderate
1-year recurrence-free survival rate	5	330	343	1.25 (0.53,2.92)	0.61	12.16	4	67	0.02	Low
3-year recurrence-free survival rate	3	288	288	2.41 (0.62,9.30)	0.20	9.84	2	80	0.07	Low
5-year recurrence-free survival rate	2	268	268	0.85 (0.16,4.46)	0.85	2.98	1	66	0.08	Low

LRH, laparoscopic repeat hepatectomy; ORH, open repeat hepatectomy; MD, mean difference; OR, odds ratio; CI, confidence interval.

#### 3.3.3 Blood transfusion

Blood transfusion data were available in six studies (23, 27–30, 32). There was no statistical difference in blood transfusion between the two groups (OR: 0.31; 95% CI:0.06 to 1.63; p = 0.17), indicating that LRH and ORH had similar effects on this item. Heterogeneity was high (I^2^ = 69%) and analyzed in the REM (**Figure 2C**).

### 3.4 Postoperative outcomes

#### 3.4.1 Overall complication rates

**Figure 3:**
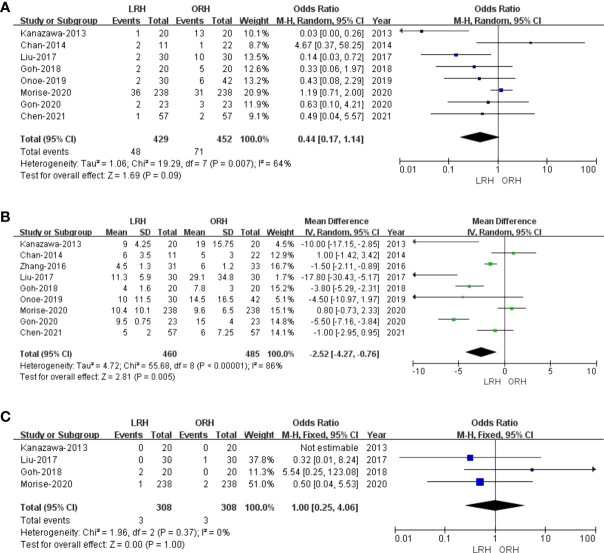
Forest plot of comparison of LRH versus ORH for postoperative outcomes of survivors. **(A)**, Forest plot for overall complication rates; **(B)**, Forest plot for the length of stay; **(C)**, Forest plot for 90-day mortality.

Eight studies (23, 27–33) with a total of 881 patients (429 and 452 underwent LRH and ORH, respectively) mentioned the overall complications, and the result of a comprehensive analysis showed that LRH was associated with a similar overall complication rate for ORH (OR: 0.44; 95% CI: 0.17 to 1.14; p = 0.09). The heterogeneity was high (I^2^ = 64%) and analyzed in the REM (**Figure 3A**).

#### 3.4.2 Length of stay

All these nine studies had reported hospitalization time. Noticeably, the meta-analysis certified that RHCC treated with LRH presented shorter hospital stay compared with the ORH group (MD = -2.52; 95% CI: -4.27 to -0.76; p = 0.005), with high heterogeneity (I^2^ = 86%) in the REM (**Figure 3B**).

#### 3.4.3 90-Day mortality

Of the nine studies, four trials (27–29, 32) performed an objective evaluation of the 90-day mortality. The result of the present study considered no difference in 90-day mortality between LRH and ORH groups (OR = 1.00; 95% CI: 0.25 to 4.06; p = 1.00), with low heterogeneity (I^2^ = 0%) in the FEM (**Figure 3C**).

### 3.5 Oncological outcomes

#### 3.5.1 Overall survival

**Figure 4:**
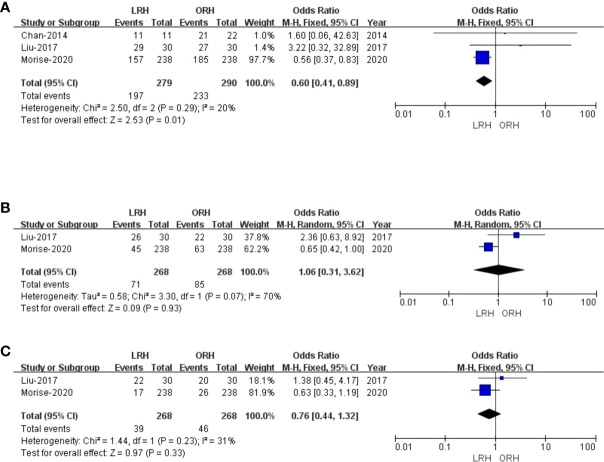
Forest plot of comparison of LRH versus ORH for the overall survival rate of survivors. **(A)**, Forest plot for 1-year overall survival time rate; **(B)**, Forest plot for 3-year survival time rate; **(C)**, Forest plot for 5-year survival time rate.

Only three studies (27, 29, 31) assessed 1-year overall survival rate, and the result of our meta-analysis demonstrated that the 1-year survival rates for LRH were lower than those for ORH (OR: 0.60; 95% CI: 0.41 to 0.89; p = 0.01), into with moderate heterogeneity (I^2^ = 20%) in the REM (**Figure 4A**). Two studies (27, 29) compared the 3-year overall survival rates, and our results revealed no difference in 3-year overall survival rate (OR: 1.06; 95% CI: 0.31 to 3.62; p = 0.93), with high heterogeneity (I^2^ = 70%) in the REM (**Figure 4B**). Two studies (27, 29) assessed the 5-year overall survival rate; similarly, LRH had a proximate 5-year overall survival rate compared with the ORH group (OR: 0.76; 95% CI: 0.44 to 1.32; p = 0.33), with moderate heterogeneity (I^2^ = 31%) in the FEM (**Figure 4C**).

#### 3.5.2 Recurrence-free survival

**Figure 5:**
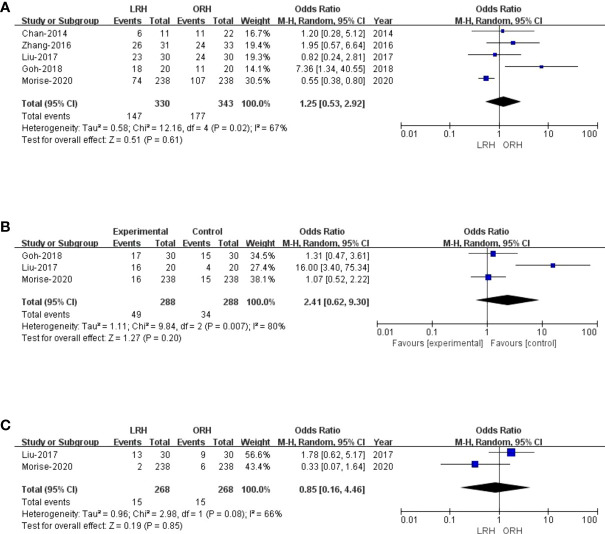
Forest plot of comparison of LRH versus ORH for the recurrence-free survival rate of survivors **(A)**, Forest plot for 1-year recurrence-free survival rate; **(b)**, Forest plot for 3-year recurrence-free survival rate; **(C)**, Forest plot for 5-year recurrence-free survival rate.

There were five studies (27–29, 31, 34) that encompassed 673 patients (330 who underwent LRH and 343 who underwent ORH) that evaluated a 1-year recurrence-free survival rate. Overall, the 1-year recurrence-free survival rate did not differ significantly between the two groups (OR: 1.25; 95% CI: 0.53 to 2.92; p = 0.61), with high heterogeneity (I^2^ = 67%) in the REM (**Figure 5A**). Three studies (27–29) reported a 3-year recurrence-free survival rate. The result of the comprehensive analysis revealed no difference in the 3-year recurrence-free survival rate between the two regimens (OR: 2.41; 95% CI: 0.62 to 9.30; p = 0.20), with high heterogeneity (I^2^ = 80%) in the REM (**Figure 5B**). Additionally, two studies (27, 29) traced a 5-year recurrence-free survival rate, and the pooled data indicated no difference in the 5-year recurrence-free survival rate between LRH and ORH groups (OR: 0.85; 95% CI: 0.16 to 4.46; p = 0.85), with low heterogeneity (I^2^ = 66%) in the FEM (**Figure 5C**).

### 3.6 Publication bias

**Figure 6:**
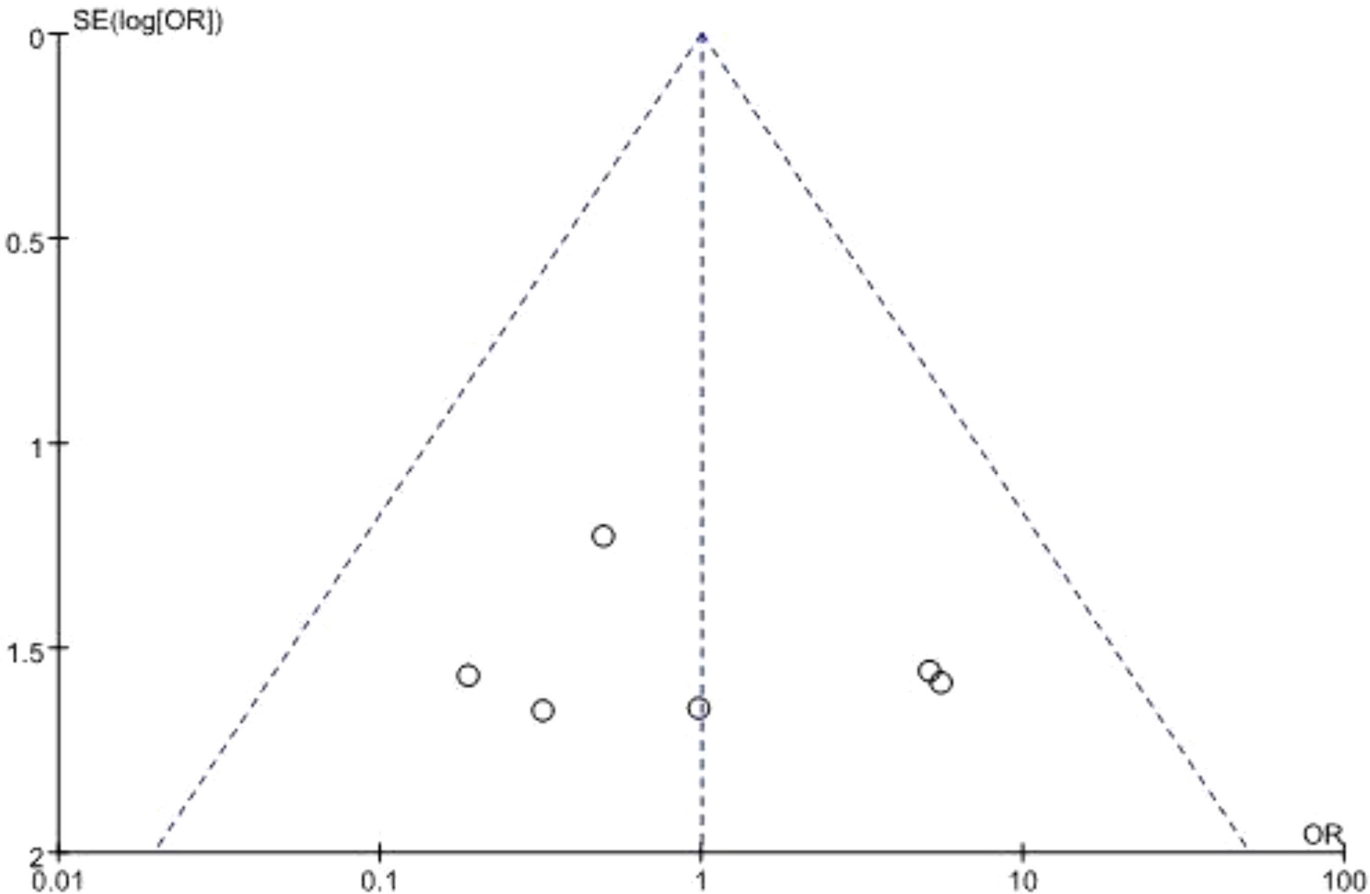
Funnel plot for publication bias.

Begg’s funnel plot was used to assess potential publication bias. All studies lie inside the 95% CIs in the funnel plot of 90-day mortality which indicated no potential publication bias (**Figure 6**).

## 4 Discussion

For the past few years, the feasibility and efficacy of LRH for RHCC compared to ORH remained ambiguous. In our latest meta-analysis of nine studies and 945 patients with post-hepatectomy HCC recurrence, we confirmed that patients with LRH had a less bleeding volume and shorter hospital stays. However, the one-year survival rate for LRH was lower than that for ORH No significant intergroup differences were observed in other operative or postoperative outcomes, with similar findings in OS and RFS.

Currently, evidence on the role of LRH in the treatment of RHCC is limited (28). Abdominal adhesions have been reported in 67%–93% of patients following abdominal surgery, particularly in patients with severe portal hypertension (30, 35). Such adhesions restricted liver mobilization and made the recognition of vital blood vessels and specific anatomical structures more difficult, which could lead to accidental vascular or biliary damage (30). Handling the serried or vascularized adhesions, especially those around the hepatic hilum or hepatoduodenal ligaments, presented manipulation challenges for LRH (27, 31). In addition to this, deformation in anatomy, formation of collateral circulation, and impaired liver function due to surgical excision of liver parenchyma may attribute to intractability in re-resection (27). Furthermore, laparoscopic resection may lead to inadequate tumor clearance due to the consideration of surgical margin (36). In particular, tumors located in the caudate lobe or seventh or eighth segment have poor visibility, angular transverse lines, and difficulty in operation limited by costal margin and dynamic diaphragms.

However, with the improvement of optical technology, the magnified view provided by laparoscopy had greatly enhanced the visual preciseness in identifying vital structures (31). Moreover, modern laparoscope cameras together with the pneumoperitoneum made the adhesion bands tense up, contributing to a more precise dissection (37). On the other hand, the positive pressure of CO_2_ pneumoperitoneum, intraoperative ultrasound, advanced transection devices, facilitation of liver inflow and outflow control, and proficient laparoscopic skills gradually lessened the uncontrollable bleeding under a laparoscope (38). Consequently, previous abdominal operations were not an absolute contraindication for the LRH (35, 39, 40). Specific selection criteria for patients performing LRH had been documented by Hu et al.: tumor located in segments 2–6, a maximum size of 5 cm, no major vessels invaded by tumors, and well-preserved liver function (41).

During the laparoscopic surgery, open techniques were used to insert the first trocar. Pneumoperitoneum was established at 12–14 mmHg, followed by insertion of remaining four to five additional trocars. Ultrasonic surgical aspiration, an ultrasonic system, and a bipolar clamp coagulation system were utilized during the operation. Resection specimens were stored in plastic bags and removed through a small incision at the umbilical site. A midline and subcostal incision was made when performing ORH procedure. A drainage tube was routinely inserted around the cut surface after operation.

Consistent with the previous meta-analyses by Peng et al. and Cai et al., we reported the advantage of LRH in bleeding volume and hospital stay over ORH. Regrettably, they enrolled only seven and six articles, including 433 and 335 patients, respectively. However, we eliminated studies comprising HCC from colorectal cancer metastasis (42–44) and replenished five pieces of literature that were published after December 2018. Furthermore, shorter hospital stay and less intraoperative blood loss were also demonstrated by Chen et al., which included 12 studies published before 1 October 2020. We included an article that was not detected by Chen. et al, as well as their PSM research. Meanwhile, five studies containing metastatic liver cancer were excluded since they violated our definition of RHCC. Through a rigorous screening and analysis process, we reached conclusions similar to those of other meta-analyses. This may be related to fewer injuries, sooner postoperative activity time, and faster bowel function recovery.

This meta-analysis comprehensively updated the security and effectiveness of LRH and ORH. However, several limitations should also be noted. Firstly, the study design of enrolled original studies was diverse, including retrospective survey, prospective study, case-match analysis, and propensity score matching (PSM). Although the PSM method can minimize selection bias and control unit balance, it will never replace randomized controlled trials on account of inherent flaws in research design. For instance, different studies performed PSM based on different potential influencing factors, and the selected factors might be inconsistent or incomplete. Besides, PSM cannot control for unknown confounders or any covariates that were either not measured or erroneously measured. In addition, retrospective studies might result in significant heterogeneity. Thus, further high-quality research is required to confirm the benefit of LRH. Secondly, the substantial heterogeneity in bleeding volume and postoperative hospital stay indicated that the conclusion should be interpreted with caution. Except for study designs, the baseline characteristics of patients, location and quantity of RHCC, surgical equipment, procedure, etc., could attribute to the heterogeneity. Thirdly, in practice, many patients were considered unsuitable for laparoscopic procedures before surgery but were then used as comparisons between laparotomy and laparoscopic interventions. However, we could not gather data about how many laparoscopic patients were deemed unfeasible. Moreover, included primary studies and our meta-analysis did not evaluate the disease’s overall burden.

There was a higher likelihood that patients undergoing LRH might previously have less complicated HCC/liver disease and resection. This selection bias should be highlighted. Finally, all of the primary research was conducted in Asia, with a particular focus on East Asia. Nevertheless, patient characteristics and diagnostic-therapeutic algorithms frequently differ from those endorsed by Western countries. Thus, we need more research from other regions, to verify the applicability of our study.

## Conclusion

Collectively, we found that LRH was likely considered a more favorable approach than ORH in specific RHCC cases for the similar risk of oncological outcomes and a quicker recovery from the procedure. However, accurate indications of LRH should be identified, and more studies are needed to reach an evidence-based conclusion.

## Data availability statement

The original contributions presented in the study are included in the article/**Supplementary Material**. Further inquiries can be directed to the corresponding author.

## Author contributions

HW conceived and designed the study. FH, HL, NL, and JL, participated in the literature search and data collection. FH, HL, and NL analyzed the data and wrote the paper. HW reviewed and edited the manuscript. All authors contributed to the article and approved the submitted version.

## Funding

This work was supported by grants from Sichuan University from 0 to 1 project (2022SCUH0017) and Sichuan Science and Technology Plan Project “International cooperation in science and technology innovation/technological innovation cooperation in Hong Kong, Macao and Taiwan” (2021YFH0095).

## Conflict of interest

All authors have completed the ICMJE uniform disclosure form.

The authors declare that the research was conducted in the absence of any commercial or financial relationships that could be construed as a potential conflict of interest.

## Publisher’s note

All claims expressed in this article are solely those of the authors and do not necessarily represent those of their affiliated organizations, or those of the publisher, the editors and the reviewers. Any product that may be evaluated in this article, or claim that may be made by its manufacturer, is not guaranteed or endorsed by the publisher.
