# Supplementary material for: Laparoscopic repeat hepatectomy versus conventional open repeat hepatectomy for recurrent hepatocellular carcinoma: A systematic review and meta-analysis
Source: Front Oncol. 2022 Sep 15;12:960204. doi: 10.3389/fonc.2022.960204 (PMC9521539; doi:10.3389/fonc.2022.960204)
Supplement: Supplementary file 1 [file DataSheet_1.docx]

**Laparoscopic repeat hepatectomy versus conventional open repeat hepatectomy for recurrent hepatocellular carcinoma: A systematic review and meta-analysis**

Fulong Hao^1,2†^, Hancong Li^3†^, Nan Li^4,5†^, Jiaxin Li ^1^, Hong Wu^1*^

^1^ Department of Liver Surgery and Liver Transplantation Centre, State Key Laboratory of Biotherapy and Cancer Center, West China Hospital, Sichuan University, Chengdu, Sichuan, CN 610041, P. R. China

^2^ Department of Hepatobiliary surgery, Suining First People's Hospital

^3^ West China School of Medicine, West China Hospital, Sichuan University, Chengdu, Sichuan, CN 610041, P. R. China

^4^Engineering Research Centre of Medical Information Technology, Ministry of Education, West China Hospital, Sichuan University, Chengdu, Sichuan, CN 610041, P. R. China

^5^Information Technology Centre, West China Hospital of Sichuan University, Chengdu, Sichuan, CN 610041P. R. China

†These authors contributed equally to this work.

***Corresponding author:**

**Hong Wu, MD, PhD,** Department of Liver Surgery and Liver Transplantation Centre, West China Hospital, Sichuan University, Chengdu, Sichuan, CN 610041, People's Republic of China. 407723080@qq.com

**Supplementary Table S1. Search strategy**

#1 (((("recurrent hepatocellular carcinoma"[MeSH Terms] OR ("recurrent "[All Fields] AND "hepatocellular carcinoma"[All Fields]) OR "recurrent liver cancer"[All Fields] OR ("recurrent"[All Fields] AND " hepatocellular cancer"[All Fields]) OR ("recurrent hepatocellular carcinoma*"[All Fields]) OR ("recurrent liver carcinoma*"[All Fields])) OR ("recurrent liver cancer*"[All Fields])) OR (RHCC)) OR (rHCC)

#2 "laparoscopic repeat hepatectomy"[MeSH Terms] OR "laparoscopic hepatectomy"[All Fields] OR "laparoscope"[All Fields] OR "laparoscopic surgery"[All Fields] OR "laparoscopic"[All Fields]

#3 "open repeat hepatectomy"[MeSH Terms] OR "open hepatectomy"[All Fields] OR "laparotomy"[All Fields] OR "open surgery"[All Fields] OR "laparotomy surgery"[All Fields]

#4 #1 AND #2 AND #3

**Supplementary Table S2. The Newcastle–Ottawa scale assessment of the included studies.**

| Study | Year | Selection | Comparability | Exposure | Total |
| --- | --- | --- | --- | --- | --- |
| Kanazawa | 2013 | 4 | 2 | 1 | 7 |
| Chan | 2014 | 4 | 2 | 1 | 7 |
| Zhang | 2016 | 4 | 2 | 2 | 8 |
| Liu | 2017 | 4 | 2 | 2 | 8 |
| Goh | 2018 | 4 | 2 | 2 | 8 |
| Onoe | 2019 | 4 | 2 | 1 | 7 |
| Morise | 2020 | 4 | 2 | 2 | 8 |
| Gon | 2020 | 4 | 2 | 2 | 8 |
| Chen | 2021 | 4 | 2 | 2 | 8 |
